# Modeling and Efficiency Analysis of a Piezoelectric Energy Harvester Based on the Flow Induced Vibration of a Piezoelectric Composite Pipe

**DOI:** 10.3390/s18124277

**Published:** 2018-12-05

**Authors:** Maoying Zhou, Mohannad Saleh Hammadi Al-Furjan, Ban Wang

**Affiliations:** 1 School of Mechanical Engineering, Hangzhou Dianzi University, Hangzhou 310027, Zhejiang, China; myzhou@hdu.edu.cn (M.Z.); rayan@hdu.edu.cn (M.S.H.A.-F.); 2 State Key Lab of Silicon Materials, Zhejiang University, Hangzhou 310027, Zhejiang, China

**Keywords:** wireless sensor network, energy harvesting, fluid structure interaction

## Abstract

This paper proposes and investigates a piezoelectric energy harvesting system based on the flow induced vibration of a piezoelectric composite cantilever pipe. Dynamic equations for the proposed energy harvester are derived considering the fluid-structure interaction and piezoelectric coupling vibration. Linear global stability analysis of the fluid-solid-electric coupled system is done using the numerical continuation method to find the neutrally stable vibration mode of the system. A measure of the energy harvesting efficiency of the system is proposed and analyzed. A series of simulations are conducted to throw light upon the influences of mass ratio, dimensionless electromechanical coupling, and dimensionless connected resistance upon the critical reduced velocity and the normalized energy harvesting efficiency. The results provide useful guidelines for the practical design of piezoelectric energy harvester based on fluid structure interaction and indicate some future topics to be investigated to optimize the device performance.

## 1. Introduction

The roaring development of engineering applications calls for more and more advanced sensors and sensor networks. However, energy supply has been a critical concern with this trend, since the capacity of currently available chemical batteries has not improved significantly in the past decades. The power requirement of wireless sensor networks, especially those located in remote applications can hardly be fulfilled. As a result, the employment of available energy in the ambient environment to replace or recharge the currently used batteries for local sensors, or the so-called energy harvesting technology, has been one the most popular research focuses over the last two decades [1]. Different ambient energy sources, such as vibration, heat, solar energy and wind energy, have been investigated and utilized in numerous energy harvesting devices with the help of smart materials and structures. Among the various proposed energy harvesting devices, piezoelectric vibration energy harvesting systems gain special focus because of their structural simplicity and compatibility with the micro electromechanical system as well as the universal presence of mechanical vibrations [2,3]. Until now, most of the proposed piezoelectric energy harvesting systems rely on the base excitation of piezoelectric structures [4,5]. The mechanisms leading to the structure vibrations and the interaction between the energy harvesting systems and the host structures are not as much concerned.

Among various mechanisms, fluid structure interaction and the resulting flow induced vibrations (FIV) have long been recognized as an origin of structural vibrations. A number of researchers [6] have exploited the interaction between structures and surrounding fluid flow to construct energy harvesting systems. Allen et al. [7] and Taylor et al. [8] firstly proposed the method of energy harvesting by placing a flexible piezoelectric composite eel in the wake of a bluff body. Beyond a critical velocity, the steady fluid flow towards the bluff body will generate alternating vortices behind the bluff body. This kind of alternating vortices will impose alternating forces upon the flexible piezoelectric composite eel and thus induce vibration in the eel, which can be used to extract energy from the fluid flow. Some other mechanisms are also incorporated into the flow energy harvesting system, such as the coupled mode flutter of airfoils, [9] the vortex-induced vibrations of rigid bodies inside steady flow, [10,11] and the galloping instabilities of flexible structures [12], etc. In addition, based on the profound research of the flapping dynamics of a flag in a uniform stream, [13,14] the utilization of compliant beams or plates to directly harvest energy from steady state fluid flow has received much attention, [15,16] with multiple aspects of the system investigated [17,18,19,20,21]. Nevertheless, nearly all the background research is based on the flow induced vibration of a piezoelectric composite beam in the externally surrounding flow, little attention is paid to the internal flow induced vibration of fluid conveying pipes, which is another popular topic in the field fluid structure interaction and flow induced vibration [22].

In this contribution, we put forward a new piezoelectric energy harvesting system based on the flow induced vibration of a piezoelectric composite pipe that conveys fluid flow. Considering the fluid-structure-electric coupling in the system, a coupled system of equations is developed to describing the dynamic behaviors of the system. Linear stability analysis with the help of numerical continuation method is made to obtain the neutrally stable mode of the system as well as the corresponding reduced flow velocity. Influences of system parameters upon the neutrally stable mode, and therefore the critical reduced flow velocity are systematically investigated. A normalized measure for the energy harvesting efficiency of the system is then developed and calculated. A series of simulations are done to figure out the parametric dependence of the proposed energy harvesting efficiency. Results are discussed with guidelines for the design of practical systems and possible extensions of the present work explored.

## 2. Problem Description and Formulation

**Figure 1:**
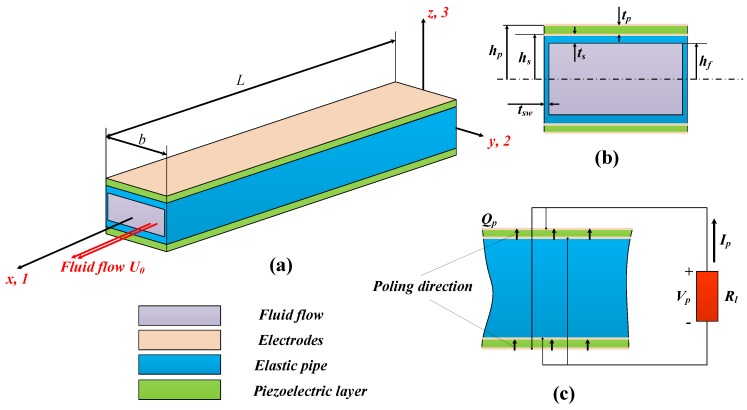
Schematic diagram of the proposed piezoelectric energy harvester based on the flow induced vibration of fluid conveying piezoelectric composite pipe: (**a**) whole view of the system, (**b**) the cross section view of the proposed energy harvester and (**c**) the externally connected circuits.

We consider here a piezoelectric composite cantilever pipe of length *L* and width *b* conveying fluid flow, as shown in Figure 1a. We set the origin of a coordinate system Oxyz to be the geometric center of the cross section of the piezoelectric composite pipe at the fixed end. The piezoelectric composite pipe consists of an elastic hollow pipe with two identical piezoelectric plates of thickness $t_{p}$ attached to its upper and lower surface. In the *z* direction, the wall thickness of the elastic pipe is $t_{s}$ while in the *y* direction, its wall thickness is $t_{sw}$. Without loss of generality, we assume in the following analysis that span-wise thickness $t_{sw}$ of the flexible is small compared to all other geometric dimensions of the elastic pipe and can be thought to be zero. Dimensions of the cross section of the piezoelectric composite pipe, which is symmetric with respect to the xOy plane, is thus shown in Figure 1b. Inner height of the flexible pipe is $2h_{f}$, which is also the height of the conveyed fluid flow. As a result, we then obtain the *z* coordinates of the upper surface of the pipe and upper surface of the piezoelectric layer as $h_{s}=h_{f}+t_{s}$ and $h_{p}=h_{s}+t_{p}$, respectively.

The two identical piezoelectric plates are connected to the external circuit in parallel [5]. They are arranged in such a way that their poling directions are aligned in the positive *z* direction, as shown in Figure 1c. For the simplicity of current analysis, the external circuit is supposed to have only a resistive load $R_{l}$. The upper and lower surface of the piezoelectric plates are covered with one single electrode, and the electric field inside the piezoelectric plates is assumed to be uniform everywhere except for the negligible edges. This is an assumption different from that of Doaré et al. [15], where a multiple electrode arrangement and its continuous limit are considered. Though there may be charge cancellation on the electrodes attached to the piezoelectric patches according to the fundamental unstable mode for the cantilever pipe, [21] we assume that the piezoelectric patches in the current contribution are covered with single electrodes for the sake of model simplicity. In the literature, there has been some research concerning the optimization of electrode distribution [23]. The consideration of the optimization of electrode distribution is a totally different topic and will not be covered here.

The proposed design of the flow energy harvesting device is distinguished in two ways. Firstly, most of the proposed flow energy harvesters in the literature [6] consider the vibration of piezoelectric composite plates or beams in surrounding flows. Here in our design, pipe structure is adopted which has not been covered in the literature. Secondly, for the pipe structure proposed, internal flow is generally used to harvest energy from fluid flow, while the conventionally used external flow is also possible to be employed. This is different from the reported flow energy harvesting devices which typically use external flow induced vibration.

**Figure 2:**
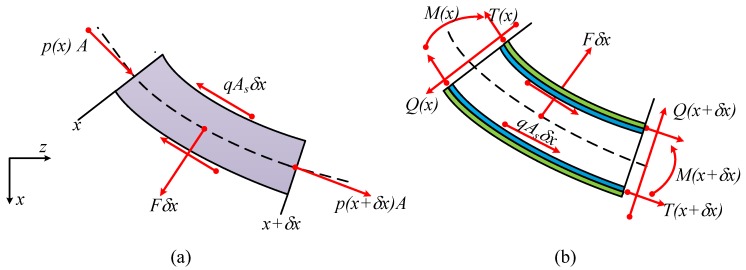
(**a**) Force diagram of the fluid element and (**b**) the piezoelectric composite pipe element.

Suppose that the flow induced vibration of the piezoelectric composite pipe is purely two dimensional in the xOz plane. The force diagram of the energy harvesting system for an arbitrary element δx of the system at the position *x* is shown in Figure 2.

According to the shown force diagram, the force balance for the fluid element [22] is described as:

(1) $$\left\{\begin{array}{cc} -A\frac{\partial p}{\partial x}-qA_{s}+F\frac{\partial w}{\partial x} & =m_{f}a_{fx},\\ -F-A\frac{\partial }{\partial x}\left(p\frac{\partial w}{\partial x}\right)-qA_{s}\frac{\partial w}{\partial x} & =m_{f}a_{fz}, \end{array}\right.$$

where *p* is the fluid pressure above the ambient atmosphere pressure, Fδx and $qA_{s}\delta x$ are the reaction forces of the piezoelectric composite pipe on the fluid in the normal and tangential direction, respectively, associated with the wall shear stress *q* at the inner wall of the piezoelectric composite pipe, $a_{fx}$ and $a_{fz}$ are the accelerations of the fluid element in the *x*- and *z*-directions, respectively, *w* is the displacement of the pipe centerline in the *z*-direction, and $m_{f}$ is the unit mass of the fluid element. Following the same routine, the force balance of piezoelectric composite pipe element (including the elastic pipe and attached piezoelectric layer) is

(2) $$\left\{\begin{array}{cc} \frac{\partial T}{\partial x}+qA_{s}-F\frac{\partial w}{\partial x} & =\left(m_{p}+m_{s}\right)a_{px},\\ \frac{\partial Q}{\partial x}+F+qA_{s}\frac{\partial w}{\partial x}+\frac{\partial }{\partial x}\left(T\frac{\partial w}{\partial x}\right) & =\left(m_{p}+m_{s}\right)a_{pz},\\ Q+\frac{\partial M}{\partial x} & =0, \end{array}\right.$$

where $a_{px}$ and $a_{pz}$ are the accelerations of the piezoelectric composite pipe element in the *x*- and *z*-directions, respectively, *T* is the longitudinal tension in the composite pipe, *Q* is the transverse shear force, *M* is the bending moment, and $m_{p}$ and $m_{s}$ are the unit length of the attached piezoelectric layers and elastic pipe, respectively.

Kinematic analysis shows that the acceleration components $a_{fx}$, $a_{fz}$, $a_{px}$, and $a_{pz}$ are

(3) $$\left\{\begin{array}{cc} a_{fx} & =\frac{\partial U_{0}}{\partial t},\\ a_{fz} & =\frac{\partial^{2}w}{\partial t^{2}}+2U_{0}\frac{\partial^{2}w}{\partial x\partial t}+U_{0}^{2}\frac{\partial^{2}w}{\partial x^{2}}+\frac{\partial U_{0}}{\partial t}\frac{\partial w}{\partial x},\\ a_{px} & =0,\\ a_{pz} & =\frac{\partial^{2}w}{\partial t^{2}}, \end{array}\right.$$

respectively, given that the vertical displacement of the pipe is much smaller compared with the pipe length and pipe height. In these expressions, $U_{0}$ is the relative velocity of the conveyed fluid to the piezoelectric composite pipe.

Combing the Equations (1), (2) and (3), we arrived at the following system of equations for the system:

(4) $$\begin{array}{cc} \left(m_{p}+m_{s}\right)\frac{\partial^{2}w}{\partial t^{2}}+m_{f}\left(\frac{\partial^{2}w}{\partial t^{2}}+2U_{0}\frac{\partial^{2}w}{\partial x\partial t}+U_{0}^{2}\frac{\partial^{2}w}{\partial x^{2}}+\frac{\partial U_{0}}{\partial t}\frac{\partial w}{\partial x}\right)-\frac{\partial^{2}M}{\partial x^{2}} & =\frac{\partial }{\partial x}\left[\left(T-pA\right)\frac{\partial w}{\partial x}\right],\\ m_{f}\frac{\partial U_{0}}{\partial t} & =\frac{\partial }{\partial x}\left(T-pA\right). \end{array}$$

Suppose that no external tension is applied to the piezoelectric composite pipe, that the gravity is neglected, and that the fluid is discharged into atmosphere with a steady velocity $U_{0}$, we have boundary conditions at the free end of the pipe as

(5) T=0,p=0atx=L.

As a result, the governing equation of the system is converted into

(6) $$\left(m_{p}+m_{s}\right)\frac{\partial^{2}w}{\partial t^{2}}+m_{f}\left(\frac{\partial^{2}w}{\partial t^{2}}+2U_{0}\frac{\partial^{2}w}{\partial x\partial t}+U_{0}^{2}\frac{\partial^{2}w}{\partial x^{2}}\right)-\frac{\partial^{2}M}{\partial x^{2}}=0.$$

By using the general Euler-Bernoulli beam theory [24], we have the the strain $S_{1}$ of the piezoelectric composite pipe in the axial direction as

(7) $$S_{1}=-z\frac{\partial^{2}w}{\partial x^{2}}.$$

The constitutive relation for the elastic pipe ($h\leq z\leq h_{s},\;or\;-h_{s}\leq z\leq -h$) is

(8) $$\sigma_{s}=E_{s}S_{1},$$

in which $\sigma_{s}$ is the related stress in the elastic pipe, $E_{s}$ is the Young’s modulus for the elastic pipe. The constitutive relation for the piezoelectric layer ($h\leq z\leq h_{s},\;or\;-h_{s}\leq z\leq -h$) is [25]

(9) $$\begin{array}{cc} \sigma_{p} & =c_{11}^{E}S_{1}-e_{31}E_{3},\\ D_{p} & =e_{31}S_{1}+\epsilon_{33}^{S}E_{3},\\ E_{3} & =-\frac{V_{p}}{t_{p}}, \end{array}$$

in which $\sigma_{p}$ is the related stress in the elastic pipe, $c_{11}^{E}$ is the elastic stiffness of the piezoelectric material at constant electric field, $e_{31}$ is the piezoelectric constant and $E_{3}$ is the electric field component in the z− direction, $\epsilon_{33}^{S}$ is the dielectric constant of the piezoelectric material at constant strain, and $V_{p}$ is the voltage developed at the piezoelectric layer. For the externally connected resistance $R_{l}$, we have according to Ohm’s law [26]:

(10) $$V_{p}+R_{l}\frac{dQ_{p}}{dt}=0,$$

where $Q_{p}$ is the amount of charge on the upper electrode of the piezoelectric layer. According to Gauss’s law [26]

(11) $$\begin{array}{cc} Q_{p} & =\int_{\Sigma }D\cdot nd\Sigma =-\int_{0}^{L}D_{p}bdx\\ & =H_{e}\int_{0}^{L}\frac{\partial^{2}w}{\partial x^{2}}dx+C_{e}V_{p}, \end{array}$$

where D is the electric displacement field inside the piezoelectric material, n is the outward unit normal for the electrodes attached to the piezoelectric patches, and

(12) $$H_{e}=2e_{31}bh_{pc},\;C_{e}=\frac{2bL\epsilon_{33}^{S}}{t_{p}},$$

and $h_{pc}$ being the coordinate the center surface of the piezoelectric layer in the vertical direction

(13) $$h_{pc}=\frac{1}{2}\left(h_{p}+h_{s}\right).$$

Combining all the information together, we obtain a system of fluid-solid-electric coupled equations for the proposed energy harvesting system:

(14) $$\begin{array}{cc} \left(m_{s}+m_{p}+m_{f}\right)\frac{\partial^{2}w}{\partial t^{2}}+2m_{f}U_{0}\frac{\partial^{2}w}{\partial t\partial x}+m_{f}U_{0}^{2}\frac{\partial^{2}w}{\partial x^{2}}+Y_{e}\frac{\partial^{4}w}{\partial x^{4}} & =0,\\ -Y_{e}\frac{\partial^{2}w}{\partial x^{2}}+H_{e}V_{p} & =M,\\ H_{e}\int_{0}^{L}\frac{\partial^{2}w}{\partial x^{2}}dx+C_{e}V_{p} & =Q_{p},\\ V_{p}+R_{l}\frac{dQ_{p}}{dt} & =0, \end{array}$$

with boundary conditions

(15) $$\begin{array}{cc} x & =0,\;w=0,\;\frac{\partial w}{\partial x}=0,\\ x & =L,\;M=0,\;\frac{\partial M}{\partial x}=0. \end{array}$$

The equations are non-dimensionalized using *L*, $L/U_{0}$, $\left(m_{s}+m_{p}\right)$, $U_{0}\sqrt{\left(m_{s}+m_{p}\right)L/C_{e}}$, and $U_{0}\sqrt{\left(m_{s}+m_{p}\right)LC_{e}}$ as characteristic length, time, mass per unit length, voltage, and charge, respectively:

(16) $$\begin{array}{cc} \left(1+M^{*}\right)\frac{\partial^{2}\bar{w}}{\partial {\bar{t}}^{2}}+2M^{*}\frac{\partial^{2}\bar{w}}{\partial \bar{t}\partial \bar{x}}+M^{*}\frac{\partial^{2}\bar{w}}{\partial {\bar{x}}^{2}}+\frac{1}{{U^{*}}^{2}}\frac{\partial^{4}\bar{w}}{\partial {\bar{x}}^{4}} & =0,\\ {\bar{V}}_{p}+\beta \frac{d{\bar{V}}_{p}}{d\bar{t}}+\frac{\alpha \beta }{U^{*}}{\left[\frac{\partial^{2}\bar{w}}{\partial \bar{x}\partial \bar{t}}\right]}_{0}^{1} & =0, \end{array}$$

with boundary conditions

(17) $$\begin{array}{cc} x & =0,\;w=0,\;\frac{\partial w}{\partial x}=0,\\ x & =1,\;\frac{\partial^{2}w}{\partial x^{2}}-\alpha U^{*}V_{p}=0,\;\frac{\partial^{3}w}{\partial x^{3}}=0, \end{array}$$

where

(18) $$M^{*}=\frac{m_{f}}{m_{s}+m_{p}},\;U^{*}=U_{0}L\sqrt{\frac{m_{s}+m_{p}}{Ye}},\;\alpha =H_{e}\sqrt{\frac{L}{C_{e}Y_{e}}},\;\beta =\frac{U_{0}R_{l}C_{e}}{L}$$

are called hereafter the mass ratio, the reduced velocity, the electromechanical coupling, and the dimensionless connected resistance, respectively.

## 3. Linear Stability Analysis

For the linear partial differential equation, we seek the stability of the trivial solution $\bar{w}\left(\bar{x},\bar{t}\right)=0$. Using the representation of eigensolution

(19) $$\left(\bar{w},{\bar{V}}_{p}\right)=Re\left\{\left(\xi ,\eta \right)e^{\sigma }\right\},$$

in which $\sigma =\sigma_{r}+j\sigma_{i}$, we have the following eigenvalue problem

(20) $$\left(1+M^{*}\right)\sigma^{2}\xi +2M^{*}\sigma \frac{\partial \xi }{\partial x}+M^{*}\frac{\partial^{2}\xi }{\partial x^{2}}+\frac{1}{{U^{*}}^{2}}\frac{\partial^{4}\xi }{\partial x^{4}}=0$$

with boundary conditions

(21) $$\begin{array}{cc} \bar{x} & =0,\;\xi =0,\;\frac{\partial \xi }{\partial \bar{x}}=0\\ \bar{x} & =1,\;\frac{\partial^{2}\xi }{\partial {\bar{x}}^{2}}+\frac{\beta \sigma }{1+\beta \sigma }\alpha^{2}\frac{\partial \xi }{\partial \bar{x}}=0,\;\frac{\partial^{3}\xi }{\partial {\bar{x}}^{3}}=0 \end{array}$$

The linear eigenvalue problem in terms of ξ, η, and σ is then solved using the “push and pull” method [27,28] with the help of the AUTO software package [29,30]. For the given values of the dimensionless parameters α, β, and $M^{*}$, the fluid-conveying piezoelectric composite pipe becomes neutrally stable at a critical reduced velocity $U_{c}^{*}$ [21]. We consider here the dependence of the critical reduced velocity $U^{*}$ upon the dimensionless electromechanical coupling α, the dimensionless connected resistance β, and the mass ratio $M^{*}$.

### 3.1. The Influence of Mass Ratio

**Figure 3:**
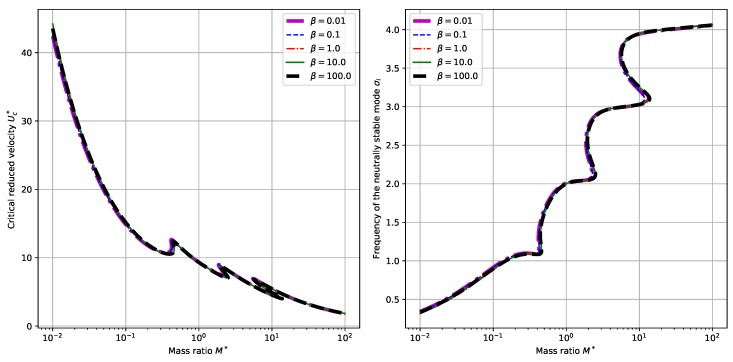
Dependence of the critical reduced velocity $U_{c}^{*}$ (**left** panel) and the corresponding neutrally stable frequency $\sigma_{i}$ (**right** panel) upon the mass ratio $M^{*}$ and different values of β. (Here we have α=0.5).

To begin with, we explore the dependence of system behaviors upon the mass ratio $M^{*}$. By setting the dimensionless electromechanical coupling α=0.5 and choosing different values for the externally connected resistance β, we can calculate the critical reduced velocity $U^{*}$ and the corresponding neutrally stable frequency $\sigma_{i}$, respectively. (Here the neutral stability means that the eigenvalue $\sigma =\sigma_{r}+j\sigma_{i}$ corresponding to the eigenmode has zero real part $\sigma_{r}=0$. Thus we denote the neutrally stable frequency of the mode by $\sigma_{i}$ hereafter.) The results are plotted in Figure 3. It is shown that in the considered range of $0.01\leq M^{*}\leq 100$, the critical reduced velocity $U_{c}^{*}$ displays an overall trend of decreasing while the neutrally stable frequency $\sigma_{i}$ displays an overall trend of increasing with the increasing of mass ratio $M^{*}$. From the perspective of energy harvesting, it is desirable for the system to have a low critical reduced velocity [16]. Therefore, we tend to choose a relatively large mass ratio $M^{*}$ in the design of related energy harvesting systems. For certain values of mass ratio $M^{*}$, there exist multiple critical velocities $U_{c}^{*}$ and corresponding neutrally stable frequencies $\sigma_{i}$. Besides, for this value of α=0.5, no big difference is found by changing the values of β from a small β=0.01 to a large β=100.0. (Actually, in both panels of Figure 3, the differences between the curves with different values of β are so small that they can hardly be discerned visually.) This can be validated by checking the Equation (17). Since the term $\frac{\beta \sigma }{1+\beta \sigma }\alpha^{2}$ occurs as a whole in the boundary conditions Equation (17), for a small value of α, say α=0.5 in our case, the variance in the values of β does not qualitatively and quantitatively change the boundary conditions Equation (17). As a result, the calculated curves for critical reduced velocities $U_{c}^{*}$ and neutrally stable frequencies $\sigma_{i}$ will not be much different.

**Figure 4:**
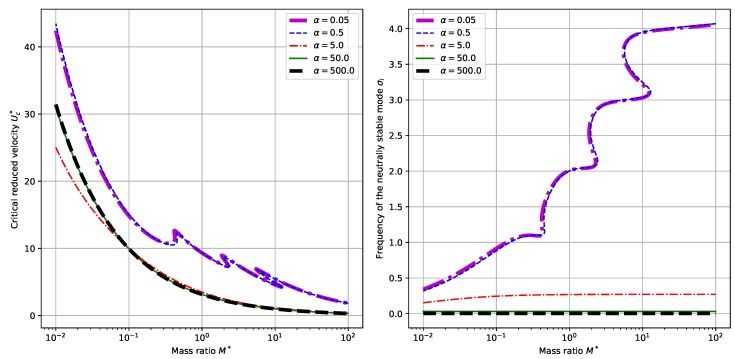
Dependence of the critical reduced velocity $U_{c}^{*}$ (**left** panel) and the corresponding neutrally stable frequency $\sigma_{i}$ (**right** panel) upon the mass ratio $M^{*}$ and different values of α. (Here we have β=1).

However, when we set α=0.5 and choose different values of α, some qualitative differences are present. The results for this set of parameter values are shown in Figure 4. When the dimensionless electromechanical coupling α is relatively small, say α=0.05 or α=0.5, there exists the same trend as that for the fixed α and varying β. While for relatively large values of α, say α=50 or α=50, no multiple correspondences of critical reduced velocities $U_{c}^{*}$ and neutrally stable frequencies $\sigma_{i}$ are shown. The critical reduced velocity $U_{c}^{*}$ is monotonically decreasing with respect to mass ratio $M^{*}$. (See the left panel of Figure 4). At the same time, the corresponding neutrally stable frequency $\sigma_{i}$ is only slightly increasing with respect to $M^{*}$. (See the right panel of Figure 4.) This kind of qualitative change with respect to α shows the influence of α upon the dynamical behaviors of the system.

### 3.2. The Influence of Electromechanical Coupling

**Figure 5:**
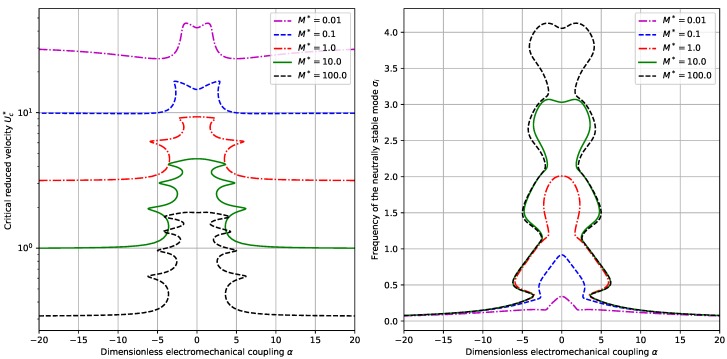
Dependence of the critical reduced velocity $U_{c}^{*}$ (**left** panel) and the corresponding neutrally stable frequency $\sigma_{i}$ (**right** panel) upon the electromechanical coupling α and different values of $M^{*}$. (Here we have β=1.0).

In this line of calculations, we first set a fixed value of β=1.0 and choose different values for $M^{*}$. The calculated critical reduced velocities $U_{c}^{*}$ and neutrally stable frequency $\sigma_{i}$ are shown in Figure 5. It is clearly shown that the value of $M^{*}$ has a large effect on the overall level of the critical reduced velocity $U_{c}^{*}$. As shown in the previous subsection, the smaller the mass ratio $M^{*}$, the larger the critical reduced velocity $U_{c}^{*}$. Note that here the value of α is allowed to be both positive and negative. This comes from the fact that in Equations (12) and (18), the term $e_{31}$ can be positive or negative for different piezoelectric materials. (Actually, for the piezoelectric material of PZT (lead zirconate titanate), $e_{31}$ is negative while for the piezoelectric material of PVDF (polyvinylidene difluoride), $e_{31}$ is positive.) Besides, from the Equation (17), we find that it is the value of $\alpha^{2}$ that affects the boundary condition and thus the dynamical system. Hence, we are interested here in the range of α in a symmetric interval. It is interesting to note that for all values of $M^{*}$, when the absolute values of α are large enough, say α>10 or α<−10 in our case, the resulting values of the critical reduced velocity $U_{c}^{*}$ and the neutrally stable frequency $\sigma_{i}$ are not much affected by the further change of α. Besides, for small values of α, say 0.5≤α≤0.5 in our case, the influences of α upon $U_{c}^{*}$ and $\sigma_{i}$ are not obvious in the sense that the values of $U_{c}^{*}$ and $\sigma_{i}$ do not vary too much. However, when α is in the intermediate range, it will largely affect the values of $U_{c}^{*}$ and $\sigma_{i}$ and a multiple correspondence is observed between the value of α and those of $U_{c}^{*}$ and $\sigma_{i}$. From the point view of energy harvesting, it will be advisable to choose either small values of α for higher neutrally stable frequencies $\sigma_{i}$ or large values of α for lower critical reduced velocities $U_{c}^{*}$.

**Figure 6:**
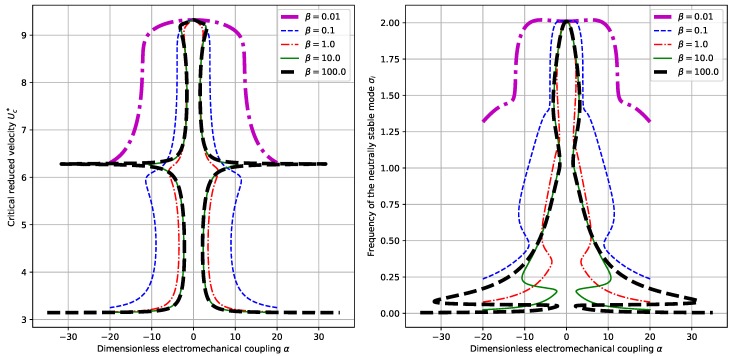
Dependence of the critical reduced velocity $U_{c}^{*}$ (**left** panel) and the corresponding neutrally stable frequency $\sigma_{i}$ (**right** panel) upon the electromechanical coupling α and different values of β. (Here we have $M^{*}=1.0$).

In the next, we set $M^{*}=1.0$ to be fixed and change the values of β, with the interesting results shown in Figure 6. In this case, the values of β influence the multiple correspondences of $U_{c}^{*}$ and $\sigma_{i}$ with respect to α. For β=0.01, no multiple correspondence is found in the range of −20≤α≤20 while for β=100.0 multiple correspondence is found for most values of α in the range −35≤α≤35. Though generally the dimensionless externally connected resistance β are determined in the design of the energy harvesting systems so that better output performances are achieved, we must note that the connection of β to the energy harvesting system does actually change the dynamical behaviors of the system. Therefore, care must be taken when deciding the parameter values of β so as not to affect such performance measures of the energy harvesting system as the operation velocity range and frequency range.

### 3.3. The Influence of Externally Connected Resistance

**Figure 7:**
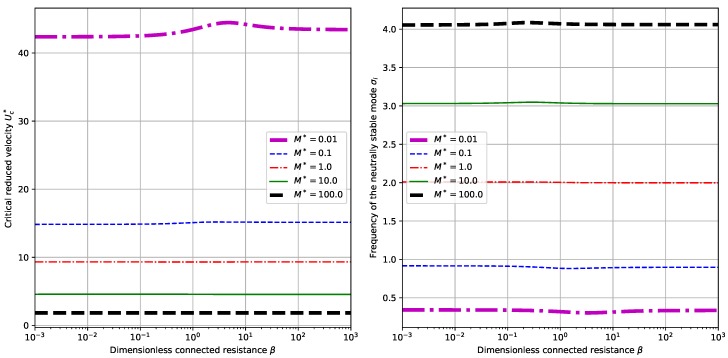
Dependence of critical reduced velocity $U_{c}^{*}$ and neutrally stable frequency $\sigma_{i}$ upon the externally connected resistance β and different values of $M^{*}$. (Here we have α=0.5).

**Figure 8:**
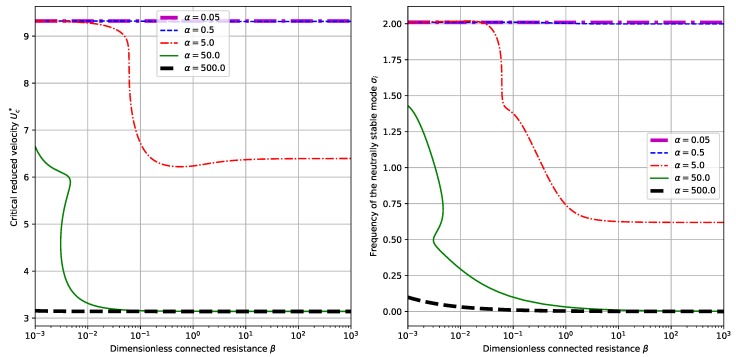
Dependence of critical reduced velocity $U_{c}^{*}$ and neutrally stable frequency $\sigma_{i}$ upon the externally connected resistance β and different values of α. (Here we have $M^{*}=1.0$).

By setting α=0.5 and choosing different values of $M^{*}$, we calculate the critical reduced velocity $U_{c}^{*}$ and the neutrally stable frequency $\sigma_{i}$ corresponding to β. The results are shown in Figure 7. For given values of $M^{*}$, the variances of $U_{c}^{*}$ and $\sigma_{i}$ with respect to β is relatively small. Nevertheless, we can find interesting peaks for each value of $M^{*}$. More interesting features are found when we fix the value of $M^{*}=1.0$ and change the values of α, for which the results are shown in Figure 8. Actually, for small values of α (say α=0.05 or α=0.5 in our case), or large values of α (say α=500.0 in our case), the variances of $U_{c}^{*}$ and $\sigma_{i}$ with respect to β are relatively small. However, for intermediate values of α, say α=5.0 and α=500.0 in our case, substantial drop is observed in $U_{c}^{*}$ and $\sigma_{i}$ when we gradually increase the value of β. From the point view of energy harvesting, it can be concluded that, larger β helps to obtain a larger operation velocity range (the critical reduced velocity $U_{c}^{*}$ is smaller) and brings about lower operation frequencies (the critical reduced velocity $\sigma_{i}$ is smaller).

## 4. Energy Harvesting Efficiency of the Proposed Device

Following the routine by Doaré et al. [15], we define the kinetic energy $E_{k}$, the elastic potential energy $E_{p}$ and electrical potential energy $E_{e}$ stored in the piezoelectric elements as

(22) $$\begin{array}{cc} E_{k} & =\int_{0}^{L}\frac{1}{2}\left(m_{s}+m_{p}\right){\left(\frac{\partial w}{\partial t}\right)}^{2}dx\\ E_{p} & =\int_{0}^{L}\frac{1}{2}Y_{e}{\left(\frac{\partial^{2}w}{\partial x^{2}}\right)}^{2}dx\\ E_{e} & =\frac{1}{2}C_{e}V_{p}^{2} \end{array}$$

respectively, while the harvested energy, or the dissipated power $P_{el}$ by the connected resistance β (suppose that all the dissipated energy by the resistance can be harvested and utilized) is

(23) $$P_{el}=-V_{p}\frac{\partial Q_{p}}{\partial t}=\frac{V_{p}^{2}}{R_{l}}$$

In the framework of linear stability analysis, it is impossible to extract any estimates of the total harvested energy. Hence we defined here a normalized measure $r_{ef}$ for the energy conversion efficiency of the system [15] by considering the total harvested energy in a working period $T_{p}$ of the system relative to the average contained energy in the system itself. That is

(24) $$r_{ef}=\frac{\int_{0}^{T_{p}}P_{el}dt}{\frac{1}{T_{p}}\int_{0}^{T_{p}}\left(E_{k}+E_{p}+E_{e}\right)dt}.$$

Using the equations indicated in [15] and considering the expression (19), the definition (24) is converted into

(25) $$r_{ef}=\frac{\frac{4\pi }{\beta \sigma_{i}}{\left|\eta \right|}^{2}}{\int_{0}^{1}\left(w_{i}^{2}{\left|\xi \right|}^{2}+\frac{1}{{U^{*}}^{2}}{\left|\frac{\partial^{2}\xi }{\partial x^{2}}\right|}^{2}\right)dx+{\left|\eta \right|}^{2}}$$

**Figure 9:**
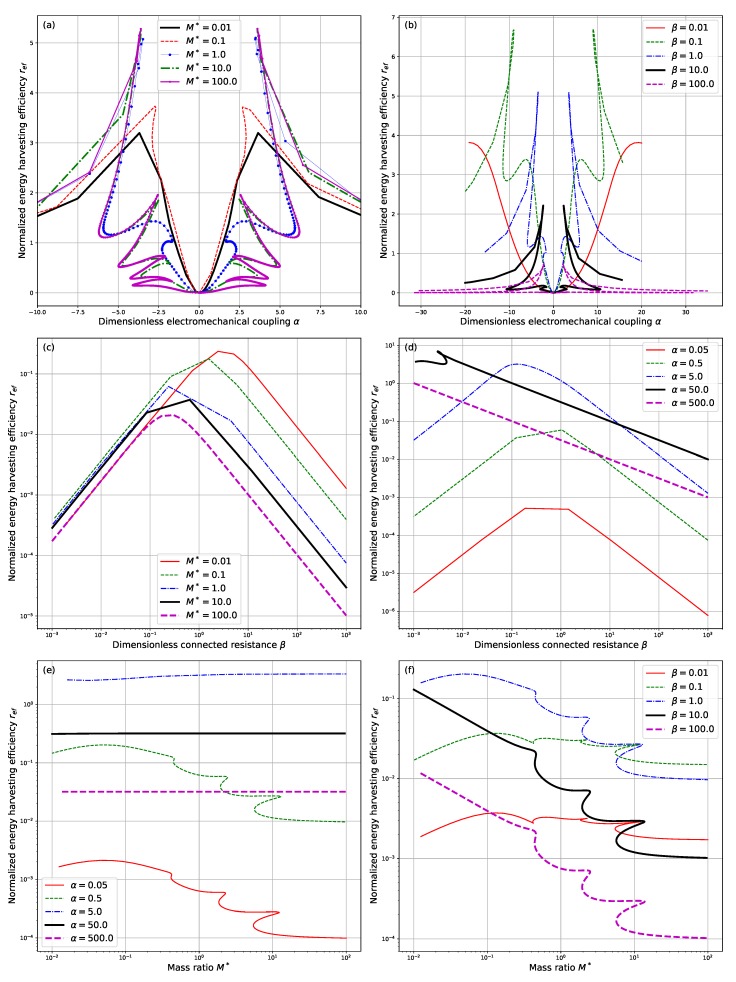
Dependence of the normalized energy harvesting efficiency $r_{ef}$ upon (**a**) the dimensionless electromechanical coupling α with β=1.0 and different values of $M^{*}$; (**b**) the dimensionless connected resistance β with α=0.5 and different values of $M^{*}$; (**c**) the mass ratio $M^{*}$ with β=1.0 and different values of α; (**d**) the dimensionless electromechanical coupling α with $M^{*}=1.0$ and different values of β; (**e**) the dimensionless connected resistance β with $M^{*}=1.0$ and different values of α; and (**f**) the mass ratio $M^{*}$ with α=0.5 and different values of β.

With different values of α, β, and $M^{*}$, the normalized energy harvesting efficiency $r_{ef}$ corresponding to the acquired neutrally stable mode is calculated according to Equation (25). The results are summarized and shown in Figure 9.

From Figure 9a,b, it is clearly shown that for different values of $M^{*}$ and β, the normalized energy harvesting efficiency $r_{ef}$ reaches its peak at some value of α. However, it should be noted that the value of α at which $r_{ef}$ is maximized is possibly subject to multiple correspondence of reduced critical velocity $U_{c}^{*}$ and neutrally stable frequency $\sigma_{i}$. This multiple correspondence actually results from the multiple correspondence of the marginally stable mode calculated to the system parameters, which has been indicated in Figure 3. This kind of multiple correspondence is prevalent in the research of dynamical systems and has been validated by many studies in the fluid structure interaction of flexible plates and steady ambient fluid flow, [14,21] where a similar dependence of critical reduced velocity and the mass ratio is observed. As a result, in practical design of the proposed energy harvesting systems, care must be taken to choose the value of electromechanical coupling α so that a better output performance is achieved. Note that for the case of $M^{*}=10.0$ and $M^{*}=100.0$, the resultant dependence curves are very close to each other and can hardly be distinguished from that shown in Figure 9a. This phenomenon lies in the fact that for a rather large value of $M^{*}$, the influence of mass ratio upon the qualitative characteristics of the devices is not so significant, which is valid according to Equation (20).

As for the influence of dimensionless connected resistance β upon the normalized energy harvesting efficiency $r_{ef}$, it is seen from Figure 9c,d, that roughly a resonance will be achieved for some values of β. This is similar to the resonance property of an oscillating RC-circuit connected to a harmonic source with frequency $\sigma_{i}$,^15^] which is indicated by Equation (16). Nevertheless, since the neutrally stable frequency $\sigma_{i}$ is dependent on dimensionless connected resistance β, a piezoelectric feedback is imposed by the externally connected circuit upon the piezoelectric composite pipe. It indicates that extra design and control strategies should be put forward to achieve a matched output performance for the externally connected circuits. However, in this way the future model must take into account the influence of the control circuit upon the whole energy harvesting system. On one hand, the dynamical behaviors of the system is partially affected as we have equivalently changed the external circuit connected to the energy harvester. On the other hand, power consumption of the control circuit is sure to affect energy harvesting efficiency of the proposed system and thus has to be minimized.

Besides, it is indicated in the previous section that the increase of mass ratio $M^{*}$ leads to the overall decrease of critical reduced velocity $U_{c}^{*}$ and the overall increase of neutrally stable frequency $\sigma_{i}$ despite the occurrence of multiple correspondence. However, here in Figure 9e,f, an overall decrease of normalized energy harvesting efficiency $r_{ef}$ is observed. It shows that we must have a trade-off between the operation velocity range and normalized energy harvesting efficiency when choosing the appropriate mass ratio $M^{*}$.

## 5. Discussion and Perspectives

The flow induced vibration of a piezoelectric composite pipe is considered here in the contribution as a candidate for flow energy harvesting. A mathematical model is derived to describe the dynamical behaviors of the proposed system by incorporating fluid induced vibration and piezoelectric coupling. Global stability analysis is done with the help of the AUTO software package. The critical reduced velocity $U_{c}^{*}$, the neutrally stable frequency $\sigma_{i}$, and the normalized energy harvesting efficiency $r_{ef}$ for each of the neutrally stable eigenmode are then calculated and summarized.

The mass ratio $M^{*}$ measures the mass per unit length conveyed in the pipe relative to that of the piezoelectric pipe. With an increasing $M^{*}$, we expect to obtain a smaller critical reduced velocity $U_{c}^{*}$ and larger neutrally stable frequency $\sigma_{i}$ but reach a lower $r_{ef}$. In practical system design, the phenomenon of multiple correspondence must be taken into account to achieve a better performance. The dimensionless electromechanical coupling α measures the coupling between the elastic pipe vibration and the circuit response. While the system behavior is only related to the absolute value of α, we can see from the simulation results that a small value of α is likely to result in larger values of $U_{c}^{*}$ and $\sigma_{i}$. At the same time, a maximum of $r_{ef}$ does exist with varying α and for this part a small value of α are not necessarily corresponding to a maximum normalized energy harvesting efficiency. The existence of multiple correspondence between α and the critical reduced velocity $U_{c}^{*}$ make things complicated. In fact, the $U_{c}^{*}$ corresponding to maximum $r_{ef}$ at a given value of α may not be achieved in practical applications since a lower $U_{c}^{*}$ also exists for the same value of α and is easier to achieve. As for the dimensionless connected resistance β, for small values of α, it affects little the values of $U_{c}^{*}$ and $\sigma_{i}$ while for larger values of α, it causes the decrease of $U_{c}^{*}$ and $\sigma_{i}$. Though a typical resonance is observed, the resonance is actually affected by the coupling between the externally connected circuit and the elastic vibration of the piezoelectric composite pipe. As a result, no quick conclusion can be made about the choice an optimal externally connected resistance β.

Compared with the piezoelectric harvesting flag proposed by Doaré et al. [15] we partially validated the obtained results [16]. First, we compare the obtained results in terms of the dependence of critical reduced velocity $U_{c}^{*}$ upon the mass ratio, which corresponds to the results shown in Figure 3 and Figure 4 and the results shown in Figure 3 of the paper by Michelin et al. [16] (In the following text, this reference paper is referred to as MD2013). The similarity of the dependence diagram results from the similar governing equations of the two systems and the similarity of instability modes. Besides, the numerical values of the the critical reduced velocity $U_{c}^{*}$ in our contribution are quite close to those in the paper MD2013. For example, in the case of α=0.5 and β=1.0, we found a $U_{c}^{*}$ of around 11 for a mass ratio of $M^{*}=\pi /8$. Note that our definition of the mass ratio $M^{*}$ is related to that defined in MD2013 by a factor of π/8. Hence, we find in Figure 3a of MD2013 that the critical reduced velocity $U_{c}^{*}$ corresponding to the case of α=0.5 and β=1 is around 12. The difference is due to the different governing equations and boundary conditions presented in the two contributions. Secondly, we compare the dependence of normalized energy harvesting efficiency $r_{ef}$ upon the dimensionless electromechanical coupling α, which corresponds to the results shown in Figure 9c,d and the results shown in Figure 13 of the paper by Michelin et al. [15]. (In the following text, this reference paper is referred to as DM2011). Again we can find similar results. In the case of β=0.5 and $M^{*}=1$ in DM2011 (In our case, it corresponds to $M^{*}=\pi /8$.), $r_{ef}$ is maximized at $\beta w_{r}\approx 1$ with a maximum of about 0.8, while in our case, the maximized $r_{ef}$ is observed to be about 0.1, corresponding to a different value of β. When we come to check the influence of α, a maximum $r_{ef}$ of 5−7 is observed in our contribution, while the maximum $r_{ef}$ is around 6 in DM2011. The closeness between the results validates our results and model.

Besides, since our model is developed in line with the method utilized in the references [15,16], and serves to theoretically investigate the feasibility and potential of flow induced energy harvesting, we define a numerical measure $r_{ef}$ of the energy conversion efficiency which is not the same as the engineering notation of efficiency. As a result, it is impossible to compare our calculated results with those presented in the literature that are obtained by experiment. However, we can find many useful insights from various publications in the field of energy harvesting. Shan et al. [31] proposed an energy harvesting system composed of two energy harvesters in a tandem configuration to improve the energy harvesting output. Song et al. [32] integrated several cylinders into the beam, which serves to amplify the flow induced vibration. Shan et al. [33] combined flow induced bending and torsion vibrations to broaden the work zones of the energy harvesters. All these proposed designs provide clues for the future modeling and optimization of our proposed design.

Though we have revealed many interesting and useful properties of the proposed piezoelectric flow induced vibration energy harvesting system, there are still open challenges. Apart from the controlling of the actual selection of neutrally stable mode and the improvement of the device efficiency, it is also interesting to explore the parameter space to acquire insights into the complex dynamical behaviors of the system, which will be helpful to the detailed design of the system in terms of its operation velocity and frequency range.

On the other hand, the coupling of fluid flow, solid vibration and electric circuits is far more complicated than the simplified version considered in this contribution. First, the fluid flow considered in this contribution is a steady state inviscid flow. However, in the general case, the fluid flow is viscous and sometimes viscoelastic. The flow velocity along the pipe is not uniform and usually of dependence upon time. Second, the dynamics of the cantilever pipe is linearized in terms of a small transverse displacement field in our analysis. For a more practical model, a motion with large displacement or even finite strain is to be considered to retain the nonlinear nature of the problem. Besides, the non-uniform distribution of electrical field and electric displacement field is to be considered to allow for the optimization of electrode patches upon the piezoelectric materials [34]. Last but not least important, the output circuit is shown to have great influence on the output performance, though we consider only a simple resistive load here. To further improve the output performance, a more complicated load circuit is to be adopted. Note that there is substantial coupling between the electric circuits and the cantilever pipe vibration. Some kind of control method is to be developed along with the adopted output circuit.
